# A meta-analytic investigation of the role of reward on inhibitory control

**DOI:** 10.1177/17470218211008895

**Published:** 2021-04-12

**Authors:** Sam Burton, Graeme Knibb, Andrew Jones

**Affiliations:** Psychological Sciences, University of Liverpool, Liverpool, UK

**Keywords:** Motivation, reward, inhibitory control, stop signal, go/no-go

## Abstract

Contemporary theories predict that inhibitory control (IC) can be improved when rewards are available for successfully inhibiting. In non-clinical samples empirical research has demonstrated some support; however, “null” findings have also been published. The aim of this meta-analysis was to clarify the magnitude of the effect of reward on IC and identify potential moderators. A total of 73 articles (contributing *k* = 80 studies) were identified from PubMed, PsycInfo, and Scopus, published between 1997 and 2020, using a systematic search strategy. A random effects meta-analysis was performed on effect sizes generated from IC tasks, which included rewarded and non-rewarded inhibition trials. Moderator analyses were conducted on clinical samples (vs “healthy controls”), task type (go/no-go vs stop signal vs Flanker vs Simon vs Stroop vs Anti-saccade), reward type (monetary vs points vs other), and age (adults vs children). The prospect of reward for successful inhibition significantly improved IC (SMD = 0.429, 95% CI = 0.288, 0.570, *I*^2^ = 96.7%) compared with no reward conditions/groups. This finding was robust against influential cases and outliers. The significant effect was present across all IC tasks. There was no evidence of the effect moderated by type of reward, age, or clinical samples. Moderator analyses did not resolve the considerable heterogeneity. The findings suggest that IC is a transient state that fluctuates in response to motivations driven by reward. Future research might examine the potential of improving IC through rewards as a behavioural intervention.

## Introduction

Inhibitory control (IC) is defined as “the (in)ability to change, suppress or delay a response that is no longer required under the current circumstances” (Logan et al., 1984) and is thought to be a core component of executive functioning and impulsive responding (Bickel et al., 2012). IC (also termed “response inhibition”) can be both reactive and proactive (Braver et al., 2007). Reactive control refers to the act of stopping a response as a “late correction” mechanism, whereas proactive control is the preplanned behavioural alterations (e.g., response slowing) in anticipation of subsequent inhibition (Aron, 2011).

Computerised tasks have been developed for the assessment and operationalisation of IC in the laboratory settings, with the most common being the “stop signal” and “go/no-go (GNG) tasks.” While these tasks measure slightly different forms of reactive IC (action cancellation vs action restraint; see Eagle et al., 2008), their component parts are similar. Both establish prepotent/dominant motor responses through promoting speeded reaction times to usually arbitrary cues. On a majority of trials, usually 75% or greater (Young et al., 2018), these responses are uninterrupted and thus prepotent or dominant responding is reinforced. However, on a minority of trials a “stop signal” or “no-go” cue is presented, prompting participants to withhold their prepotent motor response to the arbitrary cue. The inability to inhibit the prepotent response following presentation of the “stop signal” or “no-go” cue can be measured using commission errors (i.e., making a motor response to the arbitrary cue), or stop signal reaction time (SSRT: the unobserved latency of inhibition—see Band et al., 2003). Other tasks, such as the Stroop (1935) and Flanker tasks (Eriksen & Eriksen, 1974), measure the ability to override responses to congruent stimuli but are used less frequently in the literature (Diamond, 2013).

The development of these computerised tasks has led to a proliferation of studies examining IC across numerous psychological characteristics and behavioural outcomes. For example, estimates suggest that 80%–90% of self-regulation attempts require some form of inhibition (Baumeister, 2014; Hofmann et al., 2012), highlighting a key role in behavioural adaptation and human survival (Verbruggen et al., 2014). Previous research has demonstrated that effective IC is associated with increased happiness and well-being (Hofmann et al., 2014), intelligence (Polderman et al., 2009), and psychosocial functioning (Anzman-Frasca et al., 2015); while poorer IC is associated with numerous maladaptive behaviours and outcomes such as alcohol dependence (Rubio et al., 2008), incidence of overweight/obesity (Blanco-Gómez et al., 2015), poor educational attainment (Caspi et al., 2016), and crime (Vazsonyi et al., 2017).

The majority of published research considers IC as a trait-like variable, stable within individuals over long periods. However, more recent research suggests that there are both internal and external factors which might cause transient changes in stopping responses (Jones et al., 2013; Keren & Schul, 2009), which might better predict individual differences. For example, Berkman et al. (2017) propose that IC is a value-based process and represents a trade-off between short- and long-term rewards (Duckworth et al., 2016). This process involves assigning a momentary value for given behaviours, gains (e.g., money, or social approval), and costs (e.g., effort, and opportunity costs) to determine whether inhibition is required. Research has sought to enhance the “gains” valuation through the prospect of extrinsic or intrinsic rewards (Duckworth et al., 2018). This suggests that the role of motivation is key in the expression of IC processes (Poulton et al., 2016).

A number of studies have examined the role of motivation (through the prospect of obtaining rewards) on general cognitive performance, including reaction times, working memory, and task switching (Jimura et al., 2010; Umemoto & Holroyd, 2015), all of which may have a downstream influence on inhibitory processes (Miyake & Friedman, 2012; Snyder et al., 2015). Indeed, recent work has examined whether direct rewards for successful inhibition can improve IC. For example, Boehler et al. (2014) used a modified stop signal task (SST) in which the colour or the stop signal indicated whether inhibition would be rewarded or not. They demonstrated that on reward-related stop trials inhibition (measured using SSRT: the unobserved latency to inhibit behaviour) was greater than on reward-unrelated trials (see similar findings in Chiew et al., 2016; Geier & Luna, 2012; Ma et al., 2016; and Schevernels et al., 2016). In a modified GNG task (the monetary incentive delay task; Demurie et al., 2016), participants were provided information at the beginning of each trial about the magnitude of monetary rewards available (*No reward*, *Medium Reward, High Reward*). Social, as well as monetary rewards, which consisted of positive feedback (e.g., “You’re a champion” for high rewards) were also available. In this case, the effect of rewards did not influence the inhibition performance (see similar findings in Michałowski et al., 2017; Paschke et al., 2015; Schevernels et al., 2015; and Shanahan et al., 2008). Furthermore, some studies have reported the presence of reward being *detrimental* to IC (Marini et al., 2015; Williams et al., 2018; Yamaguchi & Nishimura, 2019), possibly due to a break in attentional focus caused by reward stimuli (Wang et al., 2018). Finally, studies have examined whether the presence or magnitude of reward interacts with clinical diagnoses (e.g., attention-deficit hyperactivity disorder [ADHD], substance use disorder [SUD]); however, these effects are also equivocal (Charles-Walsh et al., 2016; Chung et al., 2011; Rosell-Negre et al., 2016).

Given the considerable amount of research in the area and the inconsistent pattern of findings across individual studies, our aim was to conduct a meta-analysis on the effects of reward on IC to clarify the magnitude of effect. We also aimed to examine potential moderators of the effect, including type of task used (stop signal, GNG, Anti-saccade, Flanker, Simon, or Stroop), type of reward (monetary, points, or other), clinical samples versus non-clinical samples, and age (adults, children), in an attempt to explain potential heterogeneity of published findings. We hypothesised that the presence of rewards during IC tasks would improve subsequent IC. We did not make any directional hypotheses in regard to moderators. This meta-analysis was pre-registered on the Open Science Framework (see https://osf.io/5hbqu/) following the development of our systematic search terms, but prior to formal searches being carried out.

## Method

### Search strategy

We searched three electronic databases: Scopus, PubMed, and PsycInfo in September 2018. Searches were updated in December 2020. The following search terms were used: (1) *response inhibition* OR *inhibitory control* OR *disinhibition* OR, (2) *stop signal* OR *stroop* OR go/no* OR *flanker* OR *Anti-saccade OR simon task*, as well as (3) *reward* OR incentive*. Searches were limited to human participants, published in English, and between years 1978 and 2020. The reference list of each identified paper was examined for any eligible articles not identified through our search strategy, and this led to the addition of one further article (Asci et al., 2019).

### Eligibility criteria

Studies were eligible for the meta-analysis when the following criteria were met. First, the study had to include a validated behavioural measure of IC (outlined in Diamond, 2013), either SST, Stroop, GNG, Flanker, Anti-saccade, or Simon task. Second, the presence of reward for inhibitory performance (e.g., commission errors, SSRT, incongruent trials) was manipulated, for example, some inhibition/incongruent was rewarded, and others were not. Studies were excluded if there was a reward condition without a control (no reward condition).

### Data extraction and coding

**Figure 1.**
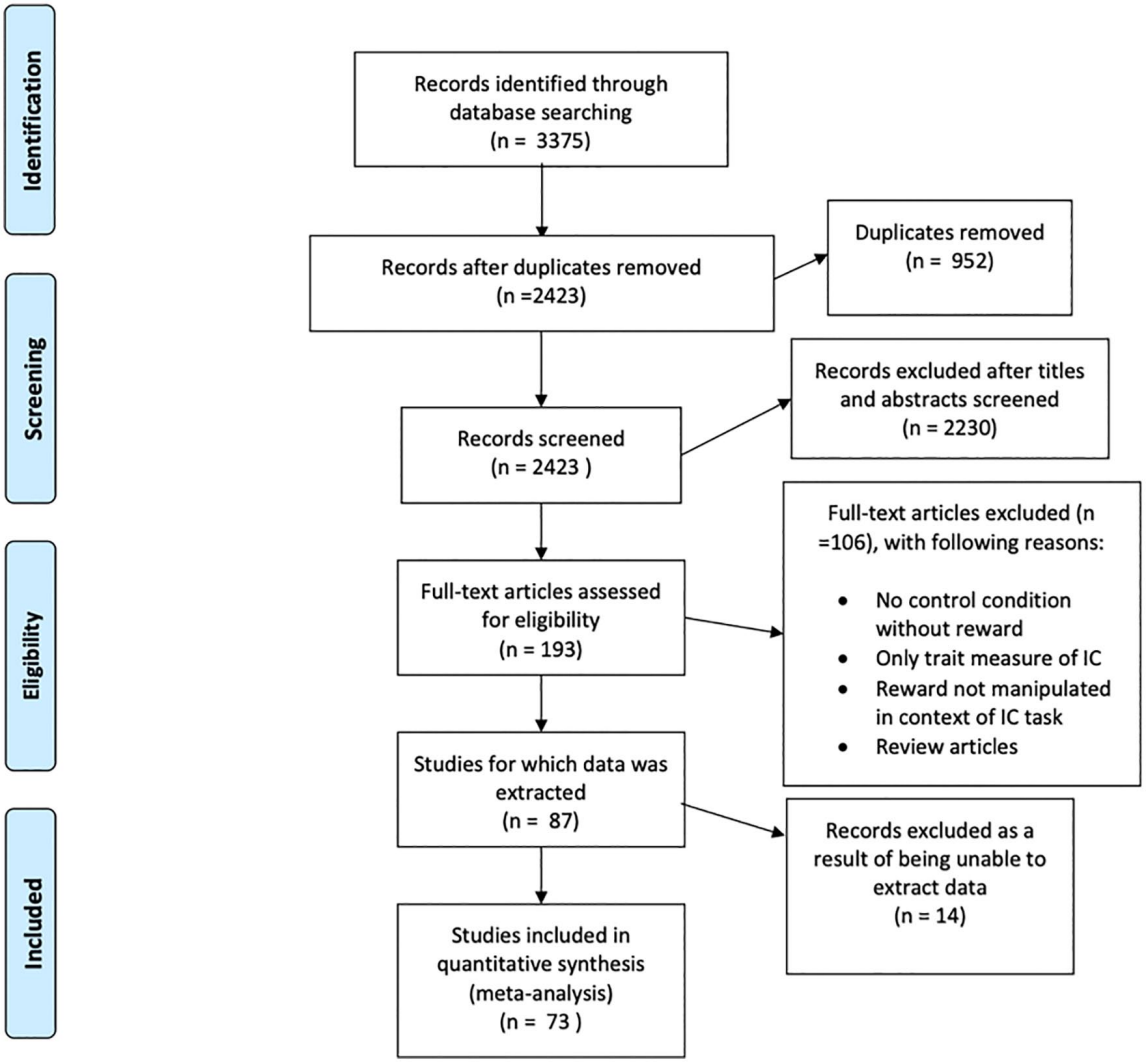
PRISMA diagram of systematic search results.

The searches yielded a total of 2,422 unique papers, an additional paper was added following reference list searches of the included articles. Titles and abstracts of these papers were examined in relation to inclusion criteria, resulting in 193 articles that were eligible for a full-text screening. Following full-text screening, 87 articles were eligible for data extraction to be used for the meta-analysis, 14 studies (16.09%) were excluded due to no reply to data requests, and 73 articles (80 effect sizes) were included. See the online supplementary material for the full table of studies included. The PRISMA flowchart can be seen in Figure 1.

### Coding of studies

S.B. and G.K. coded and extracted all 73 articles; this included sample characteristics (gender distribution, age, clinical diagnosis), methodological information (measure of IC, reward manipulation), moderator information, and IC outcome (mean RT/error rate/accuracy rate for reward and no reward condition). For the SST we extracted SSRTs; for GNG tasks we extracted error/accuracy rates; for Anti-saccade we extracted error/accuracy rates; and for the Stroop, Simon, and Flanker tasks we used incongruent RTs (as Prinzmetal et al. (2005) demonstrate, an increased sensitivity for RTs in cue-driven tasks).

Studies were coded as either adult samples, aged 18 years and above, or child samples if participants were younger than 18 years old. We examined whether studies recruited a clinical sample (e.g., ADHD, SUD, and autism spectrum disorder: see Table 1 in the supplementary material), versus “healthy controls.” Given the heterogeneity in clinical samples, we also conducted separate analyses on ADHD samples versus healthy controls, and SUD samples versus healthy controls separately.

For full-text screening, there was near-perfect agreement between reviewers (Cohen’s *k* = 0.95, *p* < .01) and substantial agreement for the data extraction stage (Cohen’s *k* = .73, *p* < .01). Any disagreements were resolved by A.J. Information about each study is presented in Table 1 in the supplementary material.

#### Data analysis

We calculated the standardised mean difference (SMD = *M*^REWARD^ − *M*^NON-REWARD^/*SD*^POOLED^) and the standard error (*SE*) of this difference, to conduct a random effects meta-analysis in “metafor” for R. We used the SMD to ensure different outcome measures used by different IC tasks and articles were comparable. For within subjects designs (e.g., Michałowski et al., 2017; Schevernels et al., 2016; Shanahan et al., 2008) the *SE* was adjusted using the correlation between the reward and control outcome (in line with the Cochrane recommendations (*SE*(SMD) = √ (1/N) + (SMD^2^/2 N) × √2(1 − correlation) (Cumpston et al., 2019). As the correlations between inhibition indices (reward and non-reward) were not readily available, we chose a correlation of .70, as recommended by previous research (Khoury et al., 2015; Rosenthal, 1991). However, we also conducted sensitivity analysis using coefficients of 0.50 and 0.90. Outliers were identified by standardising the effect sizes and examining any extreme values at a <.001 (*Z* score = ±3.30), and examining whether 95% confidence intervals did not overlap those from any other effect size. We examined potential biases in the evidence base (e.g., publication bias) using Egger’s test (Egger et al., 1997) for funnel plot asymmetry, and Trim and Fill analyses (Duval & Tweedie, 2000). We also conducted an exploratory p-curve analyses on the *p* values of the *Z* tests (SMD/*SE*), using the “dmetar” package (see supplementary analyses for p-curve figure). P-curve with a right skew (e.g., larger distribution of *p*s < .01–.025) is indicative of a likely “true” effect when the distribution of *p* values is uniformly distributed under the null hypothesis. If there is a left skew (e.g., greater distribution of *p* values between .025 and .050), this is indicative of selective reporting. Evidential value is demonstrated using the continuous and half-tests of the *pp* values (Simonsohn et al., 2015).

The meta-analysis was performed using R (R Team). Datasets and analysis script are available on OSF. Some papers reported multiple studies (e.g., Hardin et al., 2007; Padmanabhan et al., 2011; Scheres et al., 2001; Sinopoli et al., 2011), so that the primary analysis included 80 effect sizes. The degree of heterogeneity was assessed using *I*^2^. We used the following cut-offs for heterogeneity: <25% low, 25%–50% modest, and >50% high (Higgins et al., 2003). In our preregistration we stated that we would also examine proactive control; however, very few papers alluded to or measured proactive control, relative to reactive control. Therefore, we were unable to follow this up.

## Results

### Study characteristics

The majority of studies employed a within-subject (repeated measures) design, in which participants completed the measure of IC under both reward and non-reward conditions (e.g., Charles-Walsh et al., 2016; Marini et al., 2015; Scheres et al., 2001). We also identified four studies that used a between-subjects design, in which participants were randomly allocated to either the reward or non-reward condition (e.g., Huguet et al., 2004; Kohls et al., 2009; Marx et al., 2013). A number of studies examined the effect of reward on IC in clinical populations, for example, ADHD, SUD, and mental health (Byrne & Worthy, 2019; Hardin et al., 2007; Miyasaka & Nomura, 2019).

Of the studies included, the majority (78.75%) used monetary rewards (both hypothetical and real; for example, Poulton et al., 2016; Williams et al., 2018), a small number (17.50%) used “points” as rewards (e.g., Miyasaka & Nomura, 2019), and 3.75% used social rewards (e.g., Kohls et al., 2009). IC was measured using a variety of tasks. Of the 80 effect sizes, *N* = 19 (23.75%) were measured using GNG; *N* = 16 (20.00%) using SST; *N* = 13 (16.25%) using Flanker; *N* = 18 (22.50%) using Anti-saccade; *N* = 11 (13.75%) using Stroop; and *N* = 3 (3.75%) Simon task.

### Primary hypothesis: the effect of reward on IC

**Figure 2.**
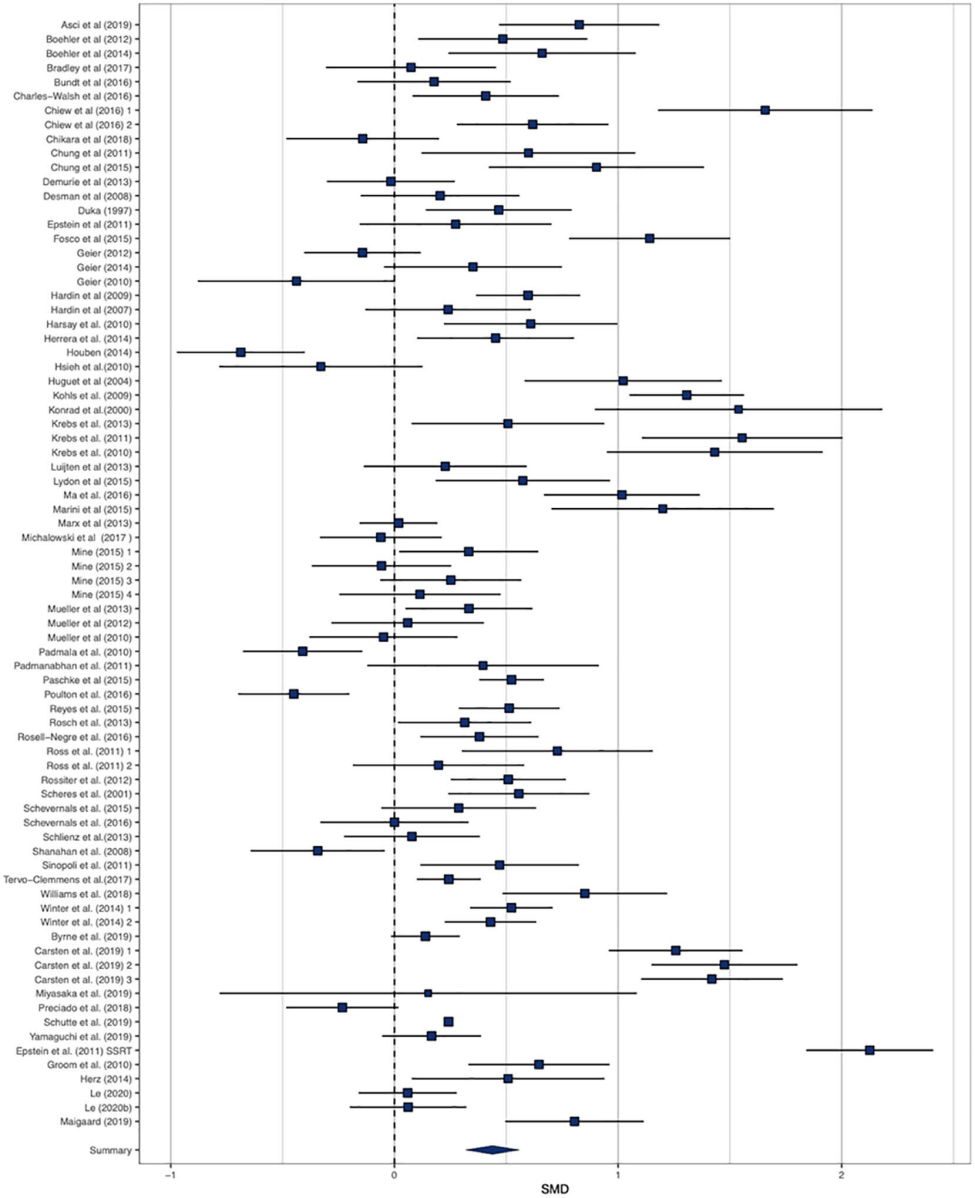
Forest plot of effect sizes for rewarded versus non-rewarded inhibitory control.

**Figure 3.**
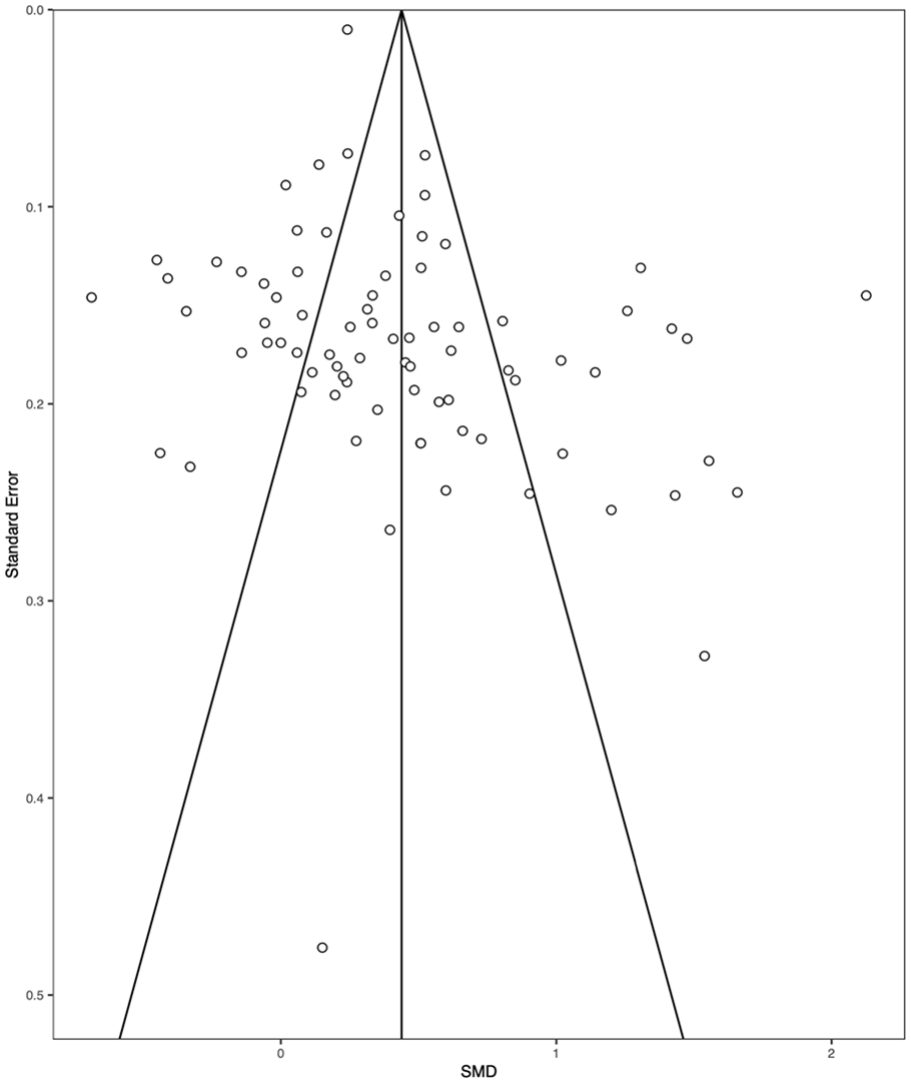
Funnel plot of the effect sizes plotted against the standard error in the meta-analysis.

Our main analysis consisted of 80 effect sizes (Figure 2). There was a small but statistically significant effect of the presence of reward improving IC (SMD = 0.429, 95% CI = [0.288, 0.570], *Z* = 5.97, *p* < .001, *I*^2^ = 96.7%). Two studies had a *Z* score ±3.30 and were removed, which did not substantially influence the effect size (SMD = .438, 95% CI = [0.319, 0.557], Z = 7.20, *p* < .001, *I*^2^ = 95.2%). A leave-one-out analysis demonstrated limited variability in the effect size (min SMD = 0.413, max SMD = 0.453: all model *p*s < .001). Trim and Fill analyses did not impute any studies, but Egger’s test of funnel plot asymmetry was significant (*Z* = 2.339, *p* = .019: see Figure 3 for funnel plot). Exploratory p-curve analyses demonstrated evidential value (full-curve *Z* = −23.98, *p* < .001 and half-curve *Z* = −20.10, *p* < .001). Sensitivity analyses demonstrated that the effect size was SMD = 0.297, 95% CI = [0.194, 0.400] if the within-subjects correlation was imputed as *r* = .50, and SMD = 0.715, 95% CI = [0.522, 0.907] if the correlation was imputed as *r* = .90. Overall, there was a small, significant effect of reward on IC, which was robust to outliers and influential cases.

### Potential moderators of the effect of reward on IC

#### Task type

Using data with outliers removed we conducted a-priori moderation on task type. There was a significant moderation effect, χ^2^(5) = 16.79, *p* = .005. There was a significant effect of reward all tasks: GNG task (*k* = 18: SMD = 0.300, 95% CI = [0.127, 0.472], *Z* = 3.407, *p* < .001, *I*^2^ = 91.25%); SST (*k* = 16: SMD = 0.410, 95% CI = [0.050, 0.770], *Z* = 2.233, *p* = .026, *I*^2^ = 95.97%); Flanker task (*k* = 13: SMD = 0.407, 95% CI = [0.130, 0.685], *Z* = 2.877, *p* = .004, *I*^2^ = 90.56%); Simon task (*k* = 3: SMD = 0.502, 95% CI = [0.126, 0.878], *Z* = 2.614, *p* = .009, *I*^2^ = 69.81%); Anti-saccade task (*k* = 18: SMD = 0.286, 95% CI = [0.128, 0.443], *Z* = 3.554, *p* < .001, *I*^2^ = 78.09%); and Stroop task (*k* = 10: SMD = 1.029, 95% CI = [0.728, 1.328], *Z* = 6.711, *p* < .001, *I*^2^ = 86.36%). The moderation effect was likely driven by the large effect sizes in Stroop tasks. Removal of the Stroop tasks from analyses made the moderator effect non-significant, χ^2^(4) = 0.986, *p* = .912. Notably, analysing the tasks separately did not substantially reduce the heterogeneity across effect sizes.

#### Age

We conducted exploratory moderation analyses on age. There were *k* = 28 effects from child samples (SMD = 0.515, 95% CI = [0.315, 0.714], *Z* = 5.053, *p* < .001, *I*^2^ = 92.18%) and *k* = 50 adult samples (SMD = 0.396, 95% CI = [0.247, 0.544], *Z* = 5.217, *p* < .001, *I*^2^ = 95.22%). There was no evidence of moderation, χ^2^(1) = 0.877, *p* = .349.

#### Reward type

We conducted exploratory moderation analysis on reward type. There were *k* = 62 effects using monetary reward (SMD = 0.392, 95% CI = [0.266, 0.518], *Z* = 6.093, *p* < .001, *I*^2^ = 94.68%), *k* = 13 effects using hypothetical “points” (SMD = 0.586, 95% CI = [0.220, 0.952], *Z* = 3.138, *p* = .002, *I*^2^ = 93.02%), and *k* = 3 effects using “other” rewards (SMD = 0.747, 95% CI = [0.208, 1.287], *Z* = 2.716, *p* = .007, *I*^2^ = 94.79%). There was no evidence of moderation, χ^2^(2) = 2.863, *p* = .239. Again, there was limited evidence that these moderator analyses reduced heterogeneity in the effect sizes.

#### Clinical samples

We conducted exploratory moderation analysis on clinical samples (vs “healthy controls”). There was no evidence of moderation, χ^2^(1) = 2.179, *p* = .140. When examining ADHD samples versus healthy controls, there was no evidence of moderation, χ^2^(1) = 0.210, *p* = .646. Similarly, when examining SUD samples versus healthy controls, there was no evidence of moderation, χ^2^(1) = 0.609, *p* = .435.

#### Supplementary analyses: statistical power of included studies

Based on the pooled effect size of SMD = 0.429, a within-subjects comparison would require 35 participants to detect this effect (one-tailed, 1–β = .80, a = .05). Of the included studies 46 (57.5%) had a large enough sample size to reliably detect this effect.

## Discussion

The current meta-analyses demonstrated that the prospect of reward can improve IC. The overall effect size was small-to-moderate, with considerable heterogeneity across the studies. Analyses indicated the effect of reward on IC was not moderated by clinical sample or type of reward used. Task type was a significant moderator of the effect of reward on IC, as the effect size was considerably larger in studies which utilised a Stroop task. The heterogeneity was not explained by any of our moderator variables.

The effect of reward on IC was consistent with recent hypotheses from theoretical models and research on healthy populations, suggesting that rewards can improve momentary IC. Specifically, we find support for value-based models (Berkman et al., 2017) in which reward appears to increase the value for a given behaviour (IC), increasing the “gain” compared with the “cost” of inhibition (Duckworth et al., 2016, 2018). The findings also support dual-process models (Evans, 2008), in which the prospect of a reward appears to improve the slower deliberate reflective systems, linked to executive control. These findings are also in line with similar meta-analyses (Jones et al., 2018), providing support for theoretical models which suggest that IC is a transient variable, which is sensitive to the internal and external factors (Jones et al., 2013; Keren & Schul, 2009).

The variability in effect sizes was not explained by clinical diagnoses in our data. This is surprising as the main clinical populations sampled were individuals with ADHD (Demurie et al., 2016; Desman et al., 2008; Ma et al., 2016) and SUD (Charles-Walsh et al., 2016; Chung et al., 2011). Both disorders are characterised by disrupted reward processing (García-García et al., 2014; Tenenbaum et al., 2018), and with this particular sensitivity to rewarding stimuli, we may have expected an enhanced effect of reward on IC for these sub-groups. In the case of SUD populations, the lack of effect of reward may be due to the severity of the condition, for example, harmful use or dependency (Byrne & Worthy, 2019), yet we did not have enough data to reliably investigate any differences by clinical diagnosis. Similarly, there was no evidence that the pooled effects were moderated by age of the participants, which may be surprising given IC improves with age into adulthood (Davis et al., 2003; Kray et al., 2020; Macdonald et al., 2014).

The effect of reward was significantly moderated by task type, with seemingly larger effects in the Stroop task. Nevertheless, reward does not appear to have a consistent effect across separate inhibitory modalities. Complex measures of IC such as the Flanker require constant monitoring and updating of rules, further complicated by manipulations of reward, requiring enhanced top-down control leading to increased working memory demand (Garon et al., 2008). IC is dependent upon the Working Memory Capacity (WMC; Burnham et al., 2014; Vandierendonck, 2014), allowing maintenance of task goals (Munakata et al., 2011), with poorer WMC and increased WMC load impairing IC (Burnham et al., 2014; Kane & Engle, 2000; Unsworth et al., 2004).

A potential mechanism by which reward improves IC may be through attentional processes. Reward may increase the detection of the inhibitory signal (particularly when the inhibitory and reward signal are the same; see Schevernels et al., 2015), leading to improved stimulus detection and reactive control (van den Berg et al., 2014; Wang et al., 2018); however, future research is needed to clarify these predictions. Research should also attempt to elucidate any individual differences which might serve to moderate the effects, for example, reward sensitivity (Capa & Bouquet, 2018). Unfortunately, we could not examine the effect of reward on reactive and proactive control due to lack of data available, therefore conclusions cannot be drawn about the mechanism that reward affects IC, for example, reactive or proactive control. Future studies should attempt to disentangle these effects to improve our overall understanding of IC (Verbruggen et al., 2014).

Given that reward appears to significantly improve IC, there are implications for the development of self-control interventions which focus on IC (e.g., inhibitory control training [ICT]). Recent meta-analyses suggest that ICT leads to short-term changes in behaviour (Allom et al., 2016; Jones et al., 2016). Reward may be used to increase the value of health-related cues (e.g., healthy foods) or devalue unhealthy behaviour-related cues (e.g., unhealthy foods) within these tasks. The opportunity to gain rewards for avoiding health risk and actively engaging in health promotion behaviour (Higgins et al., 2004; Vlaev et al., 2019) may serve to improve associative learning and strengthen the intervention effects (Schultz, 2002; Zhang et al., 2014).

We found evidence of bias in the literature following Egger’s test. While this suggests that publication bias is having a persuasive influence on the literature, researchers have suggested that such analysis is interpreted with caution, particularly when there is heterogeneity in the dataset (Shi & Lin, 2019). As such, researchers should endeavour to preregister their work to provide increased transparency. There should be particular focus on replication attempts, as meta-analytic effect sizes are proposed to be nearly three times as large as registered replications (Kvarven et al., 2019).

We acknowledge the following limitations. First, we did not assess neuropsychological outcomes (such as event-related potentials) which were presented in some of the research (Chung et al., 2011; Schevernels et al., 2015). These outcomes may be more sensitive than behavioural measures and provide a deeper understanding of the role of reward on IC, allowing the formation of a more comprehensive mechanism. Second, reward was only assessed in the form of extrinsic motivation, for example, in the presence of a reward specific cue. As such, future work should endeavour to examine the work of intrinsically rewarding appetitive stimuli to examine whether similar effects on IC are observed as described here. There is a large amount of variability in the clinical populations in the current meta-analysis, which may vary in their responsiveness to reward, making it difficult to draw conclusions on the moderating effect of clinical diagnosis on reward and IC. Therefore, interpretation of the (lack of) findings should remain cautious. Future research should seek to look at specific populations in respect to this, to better our understanding of the potential moderating role of given clinical diagnoses.

To conclude, the meta-analysis presented here suggests that the presence of reward can improve IC. Despite previous literature suggesting that individuals diagnosed with ADHD or SUDs have increased reward sensitivity, suggested a moderating role of diagnosis, we found no such evidence to support this. With reward significantly improving IC, this provides a potential avenue of treatment development for ICT, specifically producing a more prolonged behavioural change.

## Supplemental Material

sj-docx-1-qjp-10.1177_17470218211008895 – Supplemental material for A meta-analytic investigation of the role of reward on inhibitory control Click here for additional data file. Supplemental material, sj-docx-1-qjp-10.1177_17470218211008895 for A meta-analytic investigation of the role of reward on inhibitory control by Sam Burton, Graeme Knibb and Andrew Jones in Quarterly Journal of Experimental Psychology
