# Supplementary material for: A meta-analytic investigation of the role of reward on inhibitory control
Source: Q J Exp Psychol (Hove). 2021 Apr 12;74(10):1818–28. doi: 10.1177/17470218211008895 (PMC8392762; doi:10.1177/17470218211008895)

Supplementary Material for:

**A meta-analytic investigation of the role of reward on inhibitory control**

Sam Burton, Graeme Knibb, and Andrew Jones^1^

| **Study** | **N** | **Mean Age** | **Females %** | **Heavy drinkers** | **Clinical sample** | **Diagnosis** | **Substance or food** | **Design** | **DV** | | | | **Reward type** | **Hypothetical** | | | **Results** | | |  |  |  |
| --- | --- | --- | --- | --- | --- | --- | --- | --- | --- | --- | --- | --- | --- | --- | --- | --- | --- | --- | --- | --- | --- | --- |
| **Go/No-Go task** | | | | | | | | | | | | | | | | | | | | | | |
| Asci et al. (2019) | 24 | 23.42 | 66.67 | NA | NA | NA | NA | Within | No-go errors | | | Money | | No | | | R>C (reduced no-go errors in reward condition compared to control) | | | |  |  |
| Charles-Walsh (2016) | 68 | 37.96 | 29.41 | NA | Yes | Opioid dependent | NA | Within | Accuracy rate | | | Money | | No | | | R=C (no significant difference in accuracy rates) | | | |  |  |
| Demurie et al. (2016) | 127 | 11.31 | 22.11 | NA | Yes | ADHD, ASD | NA | Within | Commission errors | | | Money | | NA | | | R=C (no significant differences between conditions) | | | |  |  |
| Desman et al. (2008) | 38 | 10.27 | 0 | NA | Yes | ADHD | NA | Within | Commission errors | | | Money | | NA | | | R>C (reduced no-go errors in reward condition compared to control) | | | |  |  |
| Epstein et al. (2011a) | 13 | 8.11 | 26.89 | NA | Yes | ADHD | NA | Within | Accuracy rate | | | Money | | No | | | Not reported | | | |  |  |
| Epstein et al. (2011b) | 93 | 8.11 | 25.51 | NA | Yes | ADHD | NA | Within | Accuracy rate | | | Points | | Yes | | | R>C (significantly improved accuracy rate in reward compared to control condition) | | | |  |  |
| Groom et al. (2010) | 56 | 12.53 | 3.57 | NA | Yes | ADHD | NA | Within | Commission errors rate | | | Points | | NA | | | NA | | | |  |  |
| Kohls et al. (2009) | 48 | 10.44 | 22.34 | NA | Yes | ADHD, TBI | NA | Between | False alarm rate | | | Money, social, mixed | | No | | | R>C (significantly reduced false alarm rates in the reward compared to control condition) | | | |  |  |
| Le et al. (2020a) | 49 | 23 | 50 | NA | NA | NA | NA | Within | Accuracy rate | | | Money | | Yes | | | R<C (accuracy rate was significantly lower in the reward condition compared to control) | | | |  |  |
| Le et al. (2020b) | 35 | NA | 53.06 | NA | NA | NA | NA | Within | Accuracy rate | | | Money | | Yes | | | R<C (accuracy rate was significantly lower in the reward condition compared to control) | | | |  |  |
| Luijten et al. (2013) | 36 | 25.68 | 63.69 | NA | NA | NA | NA | Within | Accuracy rate | | | Money | | NA | | | R=C (no significant difference for accuracy rates between conditions) | | | |  |  |
| Lyndon et al. (2015) | 18 | 31.06 | 27.78 | NA | NA | NA | Smokers | Within | Accuracy rate | | | Money | | No | | | R>C (Significantly greater accuracy rate in smoking as usual group) | | | |  |  |
| Michalowski et al. (2017) | 64 | 22.18 | 78.12 | NA | NA | NA | NA | Within | Commission error rate | | | Money | | NA | | | R=C (no significant difference between conditions) | | | |  |  |
| Miyasaka et al. (2019) | 40 | 11.55 | 0 | NA | Yes | ADHD | NA | Within | Commission error rate | | | Points | | No | | | R>C (significantly reduced commission error rate in reward compared to no reward condition) | | | |  |  |
| Poulton et al. (2016) | 84 | 22.90 | 84.76 | Yes | NA | NA | NA | Within | Accuracy rate | | | Points | | NA | | | R>C (significantly improved accuracy in the reward compared to control condition) | | | |  |  |
| Rossiter et al. (2012) | 85 | 26.20 | 55.04 | Yes | NA | NA | NA | Within | Accuracy rate | | | Money | | No | | | R>C (significantly increased accuracy rates in the reward compared to control condition) | | | |  |  |
| Schevernals et al. (2016) | 21 | 25.20 | 80.95 | NA | NA | NA | NA | Within | Accuracy rate | | | Money | | No | | | R=C (no significant difference in accuracy rate across conditions) | | | |  |  |
| Schutte et al. (2019) | 49 | 23.90 | 65.56 | NA | NA | NA | NA | Within | Commission error rate | | | Money | | No | | | R>C (Commission error rates were significantly lower in the reward compared to the no reward condition) | | | |  |  |
| Winter et al. (2014) Study 1 | 77 | 8.20 | 65.00 | NA | NA | NA | NA | Within | Commission error rate | | | Toy | | Yes | | | R>C (significantly reduced error rates in the reward compared to control condition) | | | |  |  |
| Winter et al. (2014) Study 2 | 60 | 7.36 | 58.33 | NA | NA | NA | NA | Within | Commission error rate | | | Toy | | Yes | | | R>C (significantly reduced error rates in the reward compared to control condition) | | | |  |  |
| **Stop-Signal task** | | | | | | | | | | | | | | | | | | | | | | |
| Boehler et al. (2012) | 18 | 22 | 83.33 | NA | NA | NA | NA | Within | SSRT | | Money | | | | | No | R>C (shorter SSRTs in reward condition compared to control) | | | | |  |
| Boehler et al. (2014) | 16 | 22.80 | 93.75 | NA | NA | NA | NA | Within | SSRT | | Money | | | | | No | R>C (shorter SSRTs in reward condition compared to control) | | | | |  |
| Byrne & Worthy (2019) | 98 | 19.31 | 63.27 | Mild substance use | NA | NA | NA | Within | SSRT | | Money | | | | | Yes | R=C (No significant difference in SSRTs) | | | | |  |
| Chikara et al. (2018) | 20 | 23.30 | 10 | NA | NA | NA | NA | Within | SSRT | | Money | | | | | NA | R=C (No significant difference in SSRTs) | | | | |  |
| Epstein et al. (2011a) | 13 | 8.11 | 26.89 | NA | Yes | ADHD | NA | Within | SSRT | | Money | | | | | NA | R=C (SSRTs were not significantly shorter for reward compared to control) | | | | |  |
| Epstein et al. (2011b) | 93 | 8.11 | 25.51 | NA | Yes | ADHD | NA | Within | SSRT | | Points | | | | | Yes | R=C (SSRTs were not significantly shorter for reward compared to control) | | | | |  |
| Fosco et al. (2015) | 59 | 10.85 | 15.52 | NA | Yes | ADHD | NA | Within | SSRT | | Money | | | | | No | NA | | | | |  |
| Herrera et al. (2014) | 21 | 31.00 | 50 | NA | NA | NA | NA | Within | SSRT | | Money | | | | | NA | R=C (SSRTs were not significantly shorter for reward compared to control) | | | | |  |
| Houben et al. (2014) | 35 | 20.97 | 100 | NA | NA | Restrained eaters | NA | Within | SSRT | | Money | | | | | No | R=C (SSRTs were not significantly shorter for reward compared to control) | | | | |  |
| Konrad et al. (2000) | 47 | 10.44 | 22.34 | NA | Yes | ADHD, TBI | NA | Within | SSRT | | Points | | | | | NA | R=C (SSRTs were not significantly shorter for reward compared to control) | | | | |  |
| Marx et al. (2013) | 78 | 26.73 | 47.43 | NA | Yes | ADHD | NA | Between | SSRT | | Points | | | | | No | NA | | | | |  |
| Padmala et al. (2010) | 35 | 22.00 | 54.29 | NA | NA | NA | NA | Within | SSRT | | Money | | | | | No | R<C (SSRTs were significantly longer in the reward condition compared to control) | | | | |  |
| Scheres et al. (2001) | 113 | 10.48 | 25.22 | NA | Yes | ADHD, ODD | NA | Within | SSRT | | Money | | | | | No | R>C (SSRTs were significantly shorter in the reward condition compared to control) | | | | |  |
| Schevernals et al. (2015) | 20 | 25.20 | 80.95 | NA | NA | NA | NA | Within | SSRT | Points | | | | | No | | | R=C (SSRTs did not significantly differ between conditions) |  |  |  |  |
| Shanahan et al. (2008) | 55 | 11.10 | 38.18 | NA | Yes | ADHD | NA | Within | SSRT | Money | | | | | Yes | | | R=C (SSRTs did not significantly differ between conditions) |  |  |  |  |
| Sinopoli et al. (2011) | 112 | 12.44 | 47.73 | NA | Yes | ADHD, TBI | NA | Within | SSRT | Points | | | | | NA | | | R>C (SSRTs were significantly shorter in the reward compared to control condition) |  |  |  |  |
| **Flanker task** | | | | | | | | | | | | | | | | | | | | | | |
| Bradley et al. (2017) | 16 | 16.30 | 45.45 | NA | Yes | Depression/anxiety | NA | Within | RT | Money | | | | | No | | | R=C (no significant difference in RT between conditions) |  |  |  |  |
| Chiew & Braver (2016) Study 1 | 24 | 19.50 | 54.17 | NA | NA | NA | NA | Within | RT | Money | | | | | No | | | R>C (RTs in the reward condition were significantly lower than the control condition) |  |  |  |  |
| Chiew & Braver (2016) Study 2 | 24 | 20.30 | 37.50 | NA | NA | NA | NA | Within | RT | Money | | | | | No | | | R=C (no significant difference in RT between conditions) |  |  |  |  |
| Hsieh et al. (2010) | 24 | 20.60 | 50.00 | NA | NA | NA | NA | Between | RT | Money | | | | | NA | | | R=C (no significant difference in RT between conditions) |  |  |  |  |
| Marini et al. (2015) | 16 | 22.70 | 24.24 | NA | NA | NA | NA | Within | RT | Money | | | | | No | | | R>C (RTs in the reward condition were significantly lower than the control condition) |  |  |  |  |
| Mine et al. (2015) Study 1 | 26 | 37.77 | 20.20 | NA | NA | NA | NA | Within | RT | Money | | | | | NA | | | R=C (no significant difference in RT between conditions) |  |  |  |  |
| Mine et al. (2015) Study 2 | 24 | 19.40 | 58.33 | NA | NA | NA | NA | Within | RT | Money | | | | | NA | | | R=C (no significant difference in RT between conditions) |  |  |  |  |
| Mine et al. (2015) Study 3 | 24 | 21.10 | 25.00 | NA | NA | NA | NA | Within | RT | Money | | | | | NA | | | R>C (RTs in the reward condition were significantly lower than the control condition) |  |  |  |  |
| Mine et al. (2015) Study 4 | 18 | 20.70 | 38.89 | NA | NA | NA | NA | Within | RT | Money | | | | | NA | | | R=C (no significant difference in RT between conditions) |  |  |  |  |
| Paschke et al. (2015) | 125 | 25.49 | 51.20 | NA | NA | NA | NA | Within | RT | Money | | | | | No | | | R>C (RTs in the reward condition were significantly lower than the control condition) |  |  |  |  |
| Rosch et al. (2013) | 55 | 11.35 | 21.82 | NA | Yes | ADHD | NA | Within | RT | Points | | | | | No | | | NA |  |  |  |  |
| Schlienz et al. (2013) | 25 | 40.00 | 48.00 | NA | NA | NA | Smokers | Within | RT | Money | | | | | NA | | | NA |  |  |  |  |
| Williams et al. (2018) | 55 | 47.00 | 64.58 | NA | NA | NA | NA | Within | RT | Money | | | | | Yes | | | R>C (RTs in the reward condition were significantly lower than the control condition) |  |  |  |  |
| Yamaguchi et al. (2019) Study 1 | 48 | 20.44 | 66.67 | NA | NA | NA | NA | Within | RT | Money | | | | | No | | | R>C (RTs in the reward condition were significantly lower than the control condition) |  |  |  |  |
| Yamaguchi et al. (2019) Study 2 | 48 | 20.44 | 72.92 | NA | NA | NA | NA | Within | RT | Money | | | | | No | | | R>C (RTs in the reward condition were significantly lower than the control condition) |  |  |  |  |
| Yamaguchi et al. (2019) Study 3 | 48 | 20.98 | 60.42 | NA | NA | NA | NA | Within | RT | Money | | | | | No | | | R>C (RTs in the reward condition were significantly lower than the control condition) |  |  |  |  |
| **Simon task** | | | | | | | | | | | | | | | | | | |  |  |  |  |
| Bundt et al. (2016) | 20 | 22.60 | 80.00 | NA | NA | NA | NA | Within | RT | Money | | | | | No | | | R>C (RTs in the reward condition were significantly lower than the control condition) |  |  |  |  |
| Herz et al. (2014) | 14 | 23.00 | 50.00 | NA | NA | NA | NA | Within | RT | Money | | | | | No | | | R>C (RTs in the reward condition were significantly lower than the control condition) |  |  |  |  |
| Maigaard et al. (2019) | 104 | 9.67 | 23.07 | NA | NA | NA | NA | Within | RT | Money | | | | | No | | | R=C (no significant difference in RT between conditions) |  |  |  |  |
| **Stroop task** | | | | | | | | | | | | | | | | | | |  |  |  |  |
| Carsten et al. (2019) Study 1 | 46 | 18.54 | 82.61 | NA | NA | NA | NA | Within | RT | Money | | | | | Yes | | | R>C (RTs in the reward condition were significantly lower than the control condition) |  |  |  |  |
| Carsten et al. (2019) Study 2 | 45 | 19.07 | 75.56 | NA | NA | NA | NA | Within | RT | Money | | | | | Yes | | | R>C (RTs in the reward condition were significantly lower than the control condition) |  |  |  |  |
| Carsten et al. (2019) Study 3 | 46 | 18.61 | 89.13 | NA | NA | NA | NA | Within | RT | Money | | | | | Yes | | | R>C (RTs in the reward condition were significantly lower than the control condition) |  |  |  |  |
| Huguet et al. (2004) | 80 | NA | 100.00 | NA | NA | NA | NA | Between | RT | Money | | | | | NA | | | R=C (no significant difference in RT between conditions) |  |  |  |  |
| Krebs et al. (2013) | 14 | 22.60 | 71.43 | NA | NA | NA | NA | Within | RT | Money | | | | | No | | | R>C (RTs in the reward condition were significantly lower than the control condition) |  |  |  |  |
| Krebs et al. (2011) | 19 | 22.60 | 52.63 | NA | NA | NA | NA | Within | RT | Money | | | | | No | | | R>C (RTs in the reward condition were significantly lower than the control condition) |  |  |  |  |
| Krebs et al. (2010) Study 1 | 20 | 22.50 | 70.00 | NA | NA | NA | NA | Within | RT | Money | | | | | No | | | R>C (RTs in the reward condition were significantly lower than the control condition) |  |  |  |  |
| Krebs et al. (2010) Study 2 | 16 | 22.65 | 56.25 | NA | NA | NA | NA | Within | RT | Money | | | | | No | | | R>C (RTs in the reward condition were significantly lower than the control condition) |  |  |  |  |
| Ma et al. (2016) | 58 | 15.33 | 29.31 | NA | Yes | ADHD | NA | Within | RT | Money | | | | | No | | | R=C (no significant difference in RT between conditions) |  |  |  |  |
| Rossell-Negre et al. (2016) | 71 | 36.77 | 14.01 | NA | Yes | Cocaine dependent | Cocaine | Within | RT | Money | | | | | No | | | R>C (RTs in the reward condition were significantly lower than the control condition) |  |  |  |  |
| **Antisaccade task** | | | | | | | | | | | | | | | | | | |  |  |  |  |
| Chung et al. (2011) | 24 | 16.95 | 50.00 | NA | Yes | SUD | NA | Within | Error rate | Money | | | | | NA | | | R>C (Reduced error rate in reward condition compared to control) |  |  |  |  |
| Duka et al. (1997) | 24 | 29.30 | NA | NA | NA | NA | NA | Within | Accuracy rate | Money | | | | | NA | | | >C (Increased accuracy rate in reward condition compared to control) |  |  |  |  |
| Geier et al. (2012) | 106 | 17.98 | 53.77 | NA | NA | NA | NA | Within | Error rate | Points | | | | | No | | | >C (significantly reduced error rates in the reward compared to control condition) |  |  |  |  |
| Geier et al. (2014) | 34 | 35.15 | 50.00 | NA | NA | NA | Smokers | Within | Error rate | Money | | | | | No | | | R>C (significantly reduced error rates in the reward compared to control condition) |  |  |  |  |
| Geier et al. (2010) | 34 | 18.50 | 52.94 | NA | NA | NA | NA | Within | Accuracy rate | Money | | | | | NA | | | R>C (significantly increased accuracy rates in the reward compared to control condition) |  |  |  |  |
| Hardin et al. (2009) | 50 | 12.93 | 50.00 | NA | Yes | Anxiety disorder | NA | Within | Accuracy rate | Money | | | | | NA | | | R>C (significantly increased accuracy rates in the reward compared to control condition) |  |  |  |  |
| Hardin et al. (2007) | 77 | 17.67 | 48.05 | NA | Yes | Anxiety disorder, depression | NA | Within | Accuracy rate | Money | | | | | NA | | | R>C (significantly increased accuracy rates in the reward compared to control condition) |  |  |  |  |
| Harsay et al. (2010) | 56 | 51.73 | 48.05 | NA | Yes | Parkinsons | NA | Within | Accuracy rate | Money | | | | | No | | | R>C (significantly increased accuracy rates in the reward compared to control condition) |  |  |  |  |
| Mueller et al. (2013) | 63 | 15.95 | 47.62 | NA | Yes | CAH | NA | Within | Error rate | Money | | | | | No | | | R>C (significantly reduced error rates in the reward compared to control condition) |  |  |  |  |
| Mueller et al. (2012) | 46 | 11.20 | 56.52 | NA | Yes | Adopted children, with a history of neglect | NA | Within | Error rate | Money | | | | | No | | | NA |  |  |  |  |
| Mueller et al. (2010) | 43 | 13.85 | 46.51 | NA | Yes | Paediatrics with bipolar | NA | Within | Accuracy rate | Money | | | | | No | | | NA |  |  |  |  |
| Padmanabhan et al. (2011) | 30 | 20.60 | 60.00 | NA | NA | NA | NA | Within | Error rate | Money | | | | | No | | | R>C (significantly reduced error rates in the reward compared to control condition) |  |  |  |  |
| Preciado et al. (2018) | 31 | 23.96 | 77.42 | NA | NA | NA | NA | Within | Error rate | Money | | | | | No | | | R=C (no significant effect of reward on error rates) |  |  |  |  |
| Reyes et al. (2015) | 103 | 15.60 | 39.81 | NA | NA | Obese & Healthy | NA | Within | Error rate | Money | | | | | NA | | | R>C (significantly reduced error rates in the reward compared to control condition) |  |  |  |  |
| Ross et al. (2011) | 16 | 21.80 | 31.70 | NA | NA | NA | NA | Within | Error rate | Money | | | | | No | | | R>C (significantly reduced error rates in the reward compared to control condition) |  |  |  |  |
| Tervo-clemmens et al. (2017) | 116 | 12.44 | 47.43 | NA | Yes | Increased risk of SUD/ early onset SUD | NA | Within | Accuracy rate | Money | | | | | Yes | | | R>C (significantly increased accuracy rates in the reward compared to control condition) |  |  |  |  |

R, Reward; C, Control; SSRT, Stop Signal Reaction Time; SUD, Substance Use Disorder; ADHD, Attention Deficit Hyperactivity Disorder; ASD, Autistic Spectrum Disorder; TBI, Traumatic Brain Injury; CAH, Congential Adrenal Hyperplasia; ODD, Oppositional Defiant Disorde; NA, Not Reportedr

Table 1.

Supplementary Fig 1: P-curve of the significant p-values from the meta-analysis


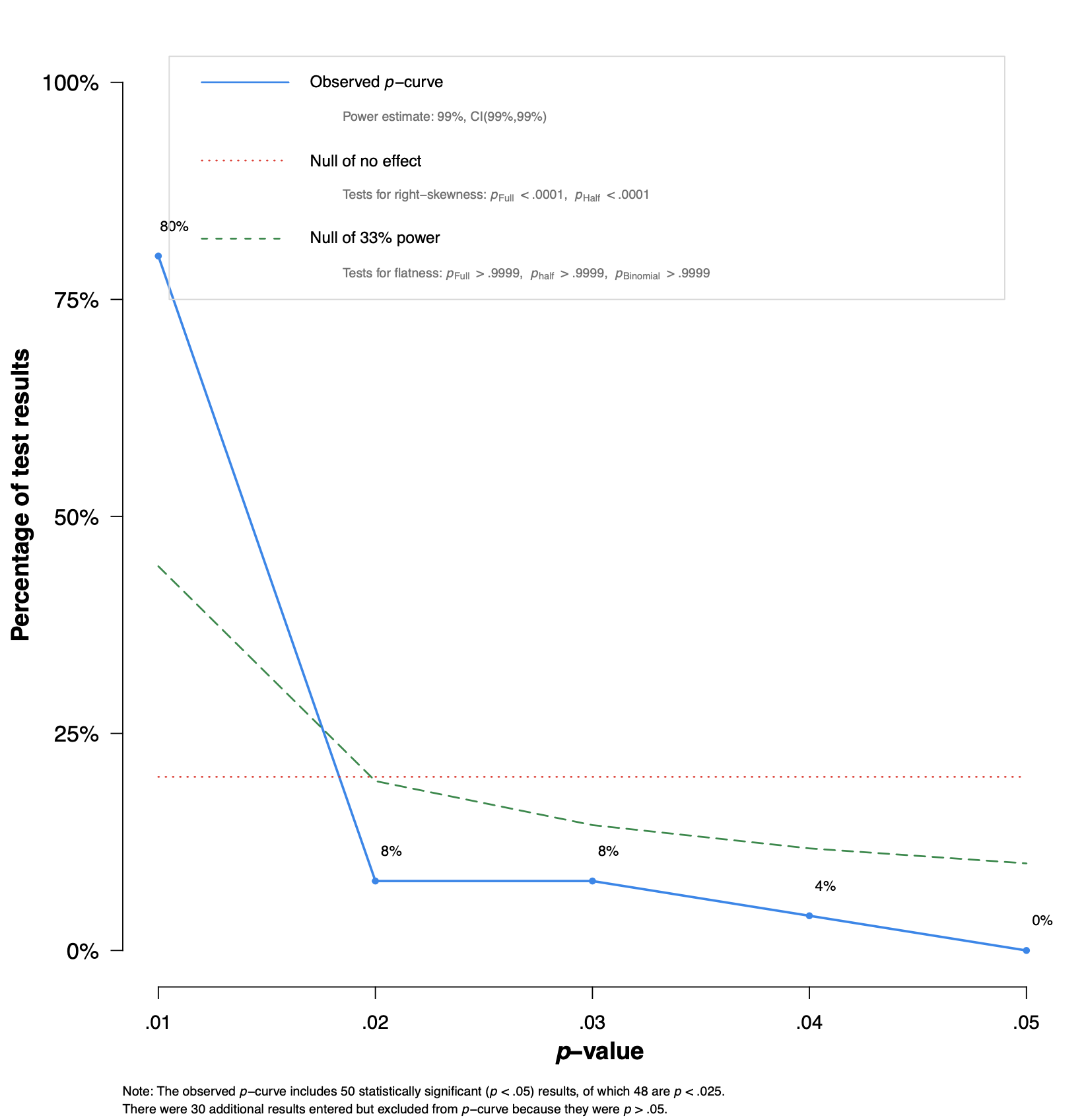

Supplement: sj-docx-1-qjp-10.1177_17470218211008895 – Supplemental material for A meta-analytic investigation of the role of reward on inhibitory control [file sj-docx-1-qjp-10.1177_17470218211008895.docx]
